# Phylogenetic Characterization of Crimean-Congo Hemorrhagic Fever Virus, Spain

**DOI:** 10.3201/eid2312.171002

**Published:** 2017-12

**Authors:** Eva Ramírez de Arellano, Lourdes Hernández, M. José Goyanes, Marta Arsuaga, Ana Fernández Cruz, Anabel Negredo, María Paz Sánchez-Seco

**Affiliations:** National Center of Microbiology Arbovirus and Imported Viral Diseases Laboratory, Madrid, Spain (E. Ramírez de Arellano, L. Hernández, A. Negredo, M. Paz Sánchez-Seco);; Gregorio Marañón Hospital, Madrid (M.J. Goyanes, A. Fernández Cruz); La Paz Hospital, IdiPaz, Madrid (M. Arsuaga)

**Keywords:** Crimean Congo hemorrhagic fever, CCFHV, ticks, *Bunyaviridae hyalomma*, zoonoses, nairovirus, phylogeny, complete genome, Spain, Africa, vector-borne infections, viruses

## Abstract

Two cases of Crimean-Congo hemorrhagic fever were reported in Spain during 2016. We obtained the virus from a patient sample and characterized its full genomic sequence. Phylogenetic analysis indicated that the virus corresponds to the African genotype III, which includes viruses previously found in West and South Africa.

Crimean-Congo hemorrhagic fever (CCHF) is a severe disease transmitted to humans mainly by ticks, primarily of *Hyalomma* spp. This zoonotic disease is caused by CCHF virus (CCHFV), a nairovirus in the family *Bunyaviridae*, which was detected first in Crimea in 1944 and 25 years later in the Democratic Republic of the Congo. Since the beginning of the 21st century, CCHFV has been spreading from disease-endemic areas to new regions previously considered free of the disease, particularly in areas where *Hyalomma* spp. ticks are present. In nature, CCHFV usually circulates between asymptomatic animals and ticks in an enzootic cycle.

CCHFV has a negative-sense and tripartite RNA genome (small [S], medium [M], and large [L] segments) with high genetic diversity. The sequences of the S segment, which is the most conserved at the nucleotide level, could be distributed in 6 lineages (*1*–*6*). Each genetic lineage has been linked to geographic regions in Africa, the Middle East, Asia, and Europe, where *Hyalomma* spp. ticks are present (*7*). Genotypes I, II, and III have been described in Africa; genotype IV in Asia; and genotypes V and VI in Europe. However, strains have moved between geographic regions; migrating birds, unregulated wildlife trade, livestock import and export, and a global movement of humans could have dispersed the virus or CCHFV-infected ticks (*8*).

In western Europe, the presence of the virus had only been detected indirectly by means of serologic methods in the serum of 2 people from southern Portugal (*9*). However, genotype III CCHFV was detected in ticks from deer captured in western Spain in 2010 and ticks from birds migrating from Morocco in 2013 (*10*,*11*). No cases among humans in Spain had been reported until 2016, when 2 autochthonous cases were diagnosed (*12*). Here, we report the complete genomic sequence of the virus from 1 of these case-patients and show the phylogenetic relationships among the 3 segments.

## The Study

We previously detected CCHFV in serum samples from the 2 patients in Spain with autochthonous CCHV (*12*); the index case-patient died. For this study, we obtained viral RNA (3.6 × 10^7^ copies/mL) from the secondary case-patient in a sample taken 4 days after onset of symptoms by using the QIAamp viral RNA Mini kit (QIAGEN, Hilden, Germany). We then amplified the virus in a single-step reverse-transcription PCR by using the SuperScript III One-Step RT-PCR system with the Platinum Taq High Fidelity DNA Polymerase kit (Invitrogen Life Technologies, Barcelona, Spain) with overlapping primers throughout the complete genome (Table). PCR conditions were amplification at 52.5°C for 30 min, 94°C for 2 min, and then 40 cycles at 94°C for 15 s, 48°C –55°C for 30 s, and 68°C for 1 min/kb, with a final extension cycle at 68°C for 5 min. We designed primers for each segment in most conserved regions after aligning available CCHFV sequences from all genotypes retrieved from GenBank (Table). We directly sequenced purified amplicons by using additional internal primers (data not shown). We assembled and analyzed the consensus sequence of each segment by using SeqMan Pro from the Lasergene Suite 12 (DNASTAR Inc., Madison, WI, USA). To get complete finished genomes, we used a hybrid-capture method as described by Blackley et al. (*13*) by using probes designed against the CCHFV sequences obtained from amplicon sequencing.

**Table:** RT-PCR primers used for generation of amplicons used for sequencing CCHFV full genome from strains isolated from patient samples in Spain, 2016*

Primer pair	5′ → 3′ sequence	5′ → 3′ position relative to reference strain AB1-2009	Amplified segment, kb	Temperature, °C†
CRCON+1	RWAAYGGRCTTRTGGAYACYTTCAC	123–147	S, 1.4	50.5
CRCON1R-S	TCTCAAAGATAYCGTTGCCGCAC	1,651–1,673		
CRCON1F-M	TCTCAAAGAAAATACTTGCGGC	1–22	M, 2.3	48
CRCON4R-M	GCATYTCAGCATCTATTGCATT	2,326–22,347		
CRCONF4F-M	TTGTAGAYCAAAGACTRGGCAG	1,775–1,796	M, 2.1	48
CRCON6R-M	GCCYGCTTCAATCAAGCTACA	3,829-3,849		
CRCON6F-M	TCAATTGAGGCACCATGGGG	3,280–3,299	M, 2.1	50
CRCON8R-M	TCTCAAAGATATAGTGGCGGCA	5,348–5,369		
CRCON1F-L	TCTCAAAGATATCAATCCCCCC	1–22	L, 2.2	48
CRCON3R-L	AGTGTCGAAAATGTRCAAATCTC	2,202--2224		
CRCON3F-L	CCTGAAAGTGACCTCACCCGC	1,562–1,582	L, 3.1	55
CRCON6R-L	TTGGCATGCTTGCAGGGCTTAG	4,656–4,677		
CRCON7F-L	TGCTTGCAGGGCTTAGTAGGCT	4,661–4,682	L, 2.3	50.5
CRCON9R-L	TCATGCATGCAACCACTGAAAT	6,948-6,969		
CCHF-L2F	GAAGAGCTATATGACATAAGGC	6,137–6,158	L, 1.6	48
CCHF-L1R	TTGGCACTATCTTTCATTTGAC	7,752–7,773		
CRCON10F-L	GGTAGTTCAGATGATTACGCAAA	7,616–7,638	L, 1.4	48
CRCON10R-L	CCTGTTAATTGTTTGCCACAA	9,019–9,039		
CRCON7200F	ATGCAACAGGTTCTGAAAAATG	7,199–7,220	L, 3.5	48
CRCON11R-L	ATGCTCCTAGTGATGCCATAATG	10,743–10,765		
CRCON12F-L	TCTTTTGAAGGTGAAGCATCTTG	10,585–10,607	L, 1.6	48
CCHF-L2R	TCTCAAAGAAATCGTTCCCCCCAC	12,149–12,172		
*F, forward; L, large segment; M, medium segment; R, reverse; RT-PCR, reverse transcription PCR; S, small segment. †Melting temperature used.				

To characterize the complete CCHFV genome, we performed a phylogenetic analysis of the full S, M, and L segments (Figure). The 3 segments were aligned by using ClustalW in MEGA 5.2 (http://www.megasoftware.net) and representative available CCHFV sequences from GenBank of all genotypes. We generated a phylogenetic tree by using neighbor-joining algorithms and analyzed 1,000 replicates for bootstrap testing. GenBank accession numbers for sequences used in this study are MF287636 for the S fragment, MF287637 for the M fragment, and MF287638 for the L fragment.

**Figure:**
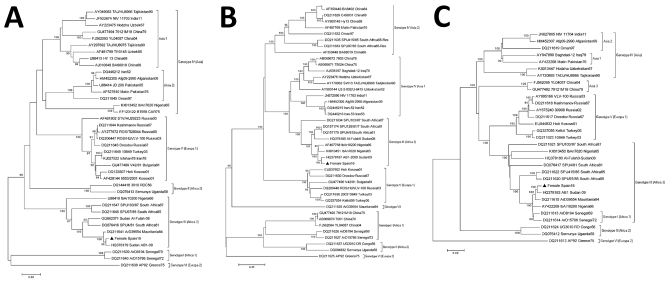
Phylogenetic analysis of Crimean-Congo hemorrhagic fever virus from patient in Spain, 2016, compared with reference sequences. A) Small segment (1,450 bp); B) medium segment (4,497 bp); C) large segment (11,829 bp). Trees were generated with the neighbor-joining method with Kimura 2-parameter distances by using MEGA version 5.1 (http://www.megasoftware.net). Bootstrap confidence limits were calculated on the basis of 1,000 replicates; numbers on branches indicate bootstrap results. Triangles indicate newly sequenced strain from Spain described in this article; other sequences are named by GenBank accession number, strain, geographic origin, and sampling year. Genotypes at right are named according to Carrol et al. (*4*); brackets indicate equivalent group nomenclature according to Chamberlain et al. (*1*). Roman numerals indicate geographic locations: I, West Africa (Africa 1); II, Central Africa (Africa 2); III, South and West Africa (Africa 3); IV, Middle East/Asia, divided into 2 groups, Asia 1 and Asia 2 (*1*); V, Europe/Turkey (Europe 1); VI, Greece (Europe 2). Scale bars indicate nucleotide substitutions per site.

The nucleotide sequence of the different CCHFV segments from the infected patient we analyzed in this study showed 99% identity with the Sudan AB1–2009 CCHFV strain (*5*) in S, M, and L segments (GenBank accession nos. HQ378179.1, HQ378187.1, and HQ378183.1, respectively) (Figure) and were grouped within genotype III (Africa 3). CCHFV found in ticks from Spain in 2010 and from birds from Morocco in 2011 also clustered in this group (*10*,*11*). In addition, no reassortant segment has been found in the analysis of the full genome, even though reassortant strains have been described in this genotype (*2*). 

## Conclusions

The results of the sequence analysis we describe corroborate our previous results (*12*), obtained by analyzing a small fragment in the S segment, showing that CCHFV from genotype III (Africa III) is circulating in southwestern Europe. CCHFV circulating in Spain caused 2 autochthonous cases that resulted in the death of the index case-patient and a serious illness in the second case-patient, providing evidence of its pathogenicity. The risk for infection in Spain is considered low, but human infection caused by the bite of an infected tick has occurred 6 years after the virus was discovered in ticks (*10*). Because the virus is circulating in Spain, additional studies will be required to establish the distribution of the virus in this country.
