# Correction: Vol. 23, No. 12

**DOI:** 10.3201/eid2406.C12406

**Published:** 2018-06

**Keywords:** errata, erratum, correction

The timing of detection of Crimean-Congo hemorrhagic fever virus in Crimea and the Democratic Republic of the Congo were unclear in Phylogenetic Characterization of Crimean-Congo Hemorrhagic Fever Virus, Spain (E. Ramírez de Arellano et al.). The article has been corrected online (https://wwwnc.cdc.gov/eid/article/23/12/17-1002_article).

